# Crystal structure of *catena*-poly[[gold(I)-μ-cyanido-[di­aqua­bis­(2-phenyl­pyrazine)­iron(II)]-μ-cyanido] dicyanidogold(I)]

**DOI:** 10.1107/S2056989019009678

**Published:** 2019-07-12

**Authors:** Olesia I. Kucheriv, Diana D. Barakhtii, Sergey O. Malinkin, Sergiu Shova, Il’ya A. Gural’skiy

**Affiliations:** a Department of Chemistry, Taras Shevchenko National University of Kyiv, Volodymyrska St. 64, Kyiv 01601, Ukraine; b UkrOrgSyntez Ltd, Chervonotkatska St., 67, Kyiv 02094, Ukraine; c Department of Inorganic Polymers, "Petru Poni" Institute of Macromolecular Chemistry, Romanian Academy of Science, Aleea Grigore Ghica Voda 41-A, Iasi 700487, Romania

**Keywords:** crystal structure, polymeric complex, iron(II) complex, 2-phenyl­pyrazine, di­cyano­aurate, aurophillic inter­actions, offset π–π inter­actions, supra­molecular metal–organic framework

## Abstract

In the title polymeric complex, {[Fe(CN)_2_(C_10_H_8_N_2_)_2_(H_2_O)_2_][Au(CN)_2_]}_*n*_, the Fe^II^ ion, which is located on a twofold rotation axis, has a slightly distorted FeN_4_O_2_ octa­hedral geometry. It is coordinated by two phenyl­pyrazine mol­ecules, two water mol­ecules and two di­cyano­aurate anions, the Au atom also being located on a second twofold rotation axis. In the crystal, the coordinated di­cyano­aurate anions bridge the Fe^II^ ions to form polymeric chains propagating along the *b*-axis direction. In the crystal, the chains are linked by O_water_—H⋯N_di­cyano­aurate anions_ hydrogen bonds and aurophillic inter­actions [Au⋯Au = 3.5661 (3) Å], forming layers parallel to the *bc* plane. The layers are linked by offset π–π stacking inter­actions [inter­centroid distance = 3.643 (3) Å], forming a supra­molecular metal–organic framework.

## Abstract 2

Cyanide anions bridge Fe^II^ and Au^I^ cations to form a one-dimensional polymeric compound with free di­cyano­aurate anions.

## Chemical context   

The design of functional materials based on coordination compounds is an important area of current scientific research. For example, metal–organic frameworks (MOFs), which consist of metal ions and organic ligand linkers, are studied intensively. Fe-based coordination polymers with N-donor bridging ligands are well known as compounds with switchable spin states (Niel *et al.*, 2003 ▸; Gural’skiy *et al.*, 2016 ▸; Kucheriv *et al.*, 2016 ▸). This phenomenon is called spin crossover and can be observed in complexes of 3*d* ^4^–3*d* ^7^ metal ions. Applying external stimuli, such as temperature, pressure, magnetic field, light irradiation or adding a guest can affect this kind of the compound and change their properties significantly (Gütlich & Goodwin, 2004 ▸). The synthesis and crystallographic characterization of these complexes are of current inter­est because of the bis­tability of their magnetic, electrical, mechanical and optical properties (Senthil Kumar & Ruben, 2017 ▸). The parameters of these transitions could be controlled through a wide variety of available organic ligands and co-ligands. Complexes with metallo­cyanate bridges as co-ligands to N-bridging ligands form one of the largest family of spin-crossover compounds (Muñoz & Real, 2011 ▸). Here we report on a new one-dimensional polymeric compound that is similar in its structure to switchable cyano­metallates. It employs 2-phen­yl­pyrazine as a ligand and Au(CN)^2−^ as co-ligands, while coordinated H_2_O mol­ecules stabilize the Fe^II^ ions in the high-spin state.
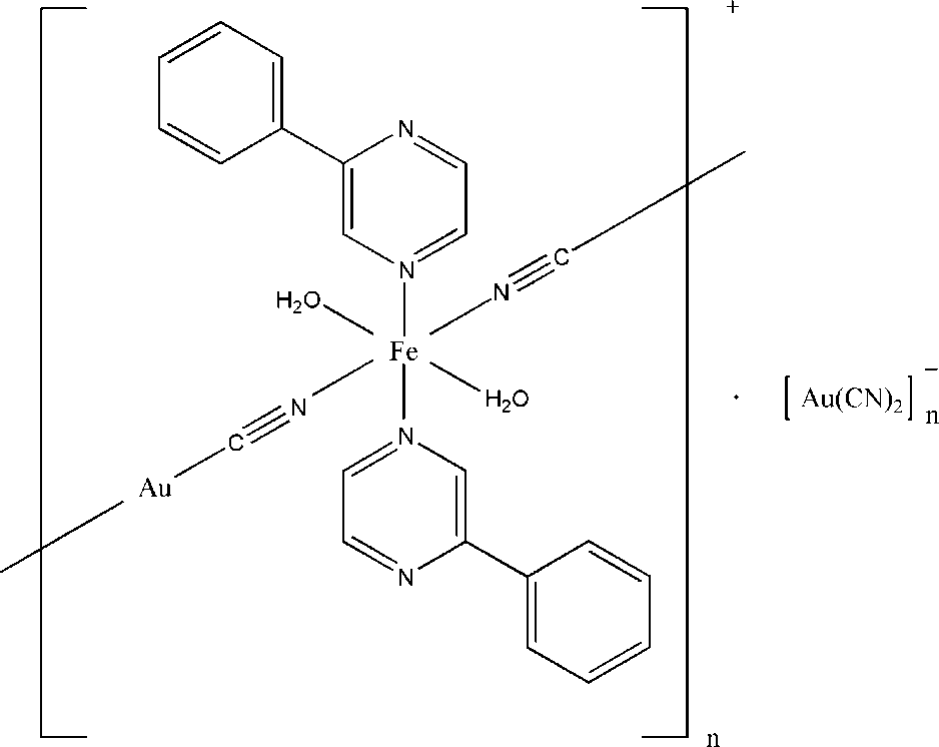


## Structural commentary   

**Figure 1:**
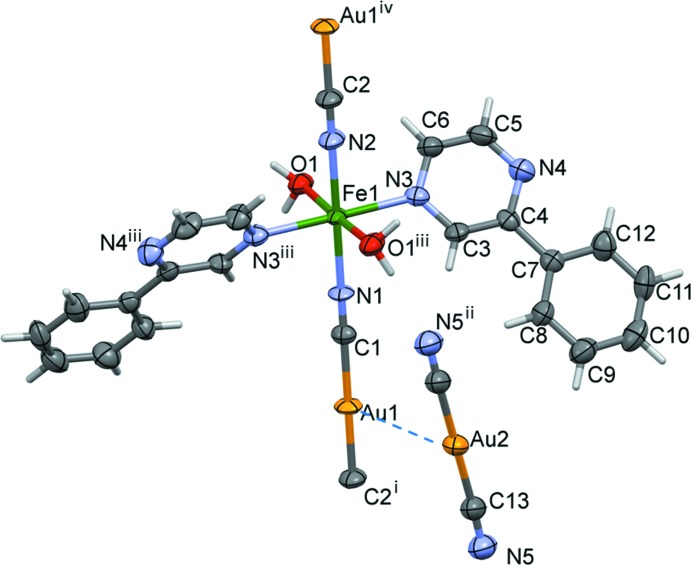
A fragment of the mol­ecular structure of the title compound, with the atom labelling Displacement ellipsoids are drawn at the 50% probability level. The Au1⋯Au2 inter­action [3.5661 (3) Å] is shown as a dashed line. [Symmetry codes: (i) *x*, *y* − 1, *z*; (ii) *x* − 1, *y* − 1, *z* − 1; (iii) −*x* − 1, *y*, −*z* + 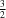; (iv) *x*, *y* + 1, *z*].

The structure of the title compound features a one-dimensional chain motif that runs parallel to the crystallographic *b* axis (Figs. 1 ▸ and 2 ▸). The compound crystallizes in the monoclinic space group *C2/c*. Selected bond distances and bond angles are given in Table 1 ▸. The coordination sphere of the Fe^II^ cation, atom Fe1, which is located on a twofold rotation axis, has a distorted octa­hedral environment [FeN_4_O_2_]. It includes two 2-phenyl­pyrazine N atoms [Fe1—N3 = 2.223 (5) Å] in axial positions, and two N atoms of cyano bridges and two water O atoms of water mol­ecules [Fe1—O1 = 2.122 (4) Å] in equatorial positions. The two CN^−^ anions bridge the Fe^II^ and Au^I^ cations [Fe1⋯Au1 = 5.244 (3) Å] to form a one-dimensional polymeric structure with bond lengths Fe1—N1 = 2.107 (5) Å and Fe1—–N2 = 2.117 (6) Å (Fig. 1 ▸ and Table 1 ▸). The Fe^II^ octa­hedral distortion parameter (the sum of the moduli of the deviations from 90° for all *cis*-bond angles) is Σ|90 - Θ| = 8.53°, where Θ are the *cis*-N—Fe—O and *cis*-N—Fe—N angles in the coordination environment of the Fe^II^ atom.

## Supra­molecular features   

**Figure 2:**
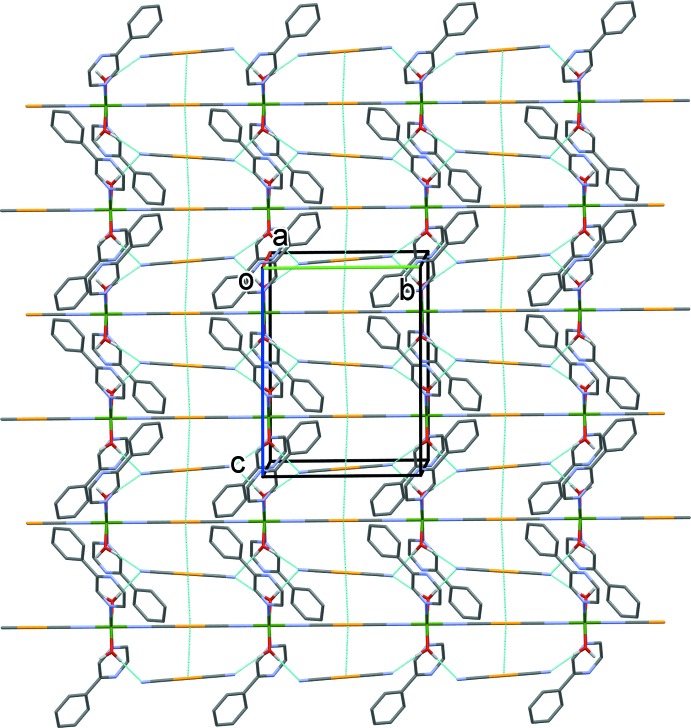
A view along the *a* axis of the crystal packing of the title compound. The hydrogen bonds (Table 2 ▸) and aurophillic inter­actions as shown as dashed lines. For clarity, the C-bound H atoms have been omitted.

The crystal packing features different types of weak inter­actions (see Table 2 ▸ and Figs. 2 ▸ and 3 ▸). The free di­cyano­aurate anions are linked to the polymeric chains by O_water_—H⋯N hydrogen bonds [O1—H1*A*⋯N5^v^ = 2.02 Å and O1—H1*B*⋯N5^vi^ = 2.18 Å; Table 2 ▸], and by aurophillic inter­actions [Au1⋯Au2 = 3.566 (2) Å], forming layers parallel to the *bc* plane. The layers are then linked *via* offset π–π inter­actions involving a pyrazine ring as an acceptor and a phenyl ring as a donor of electron density, forming a supra­molecular metal–organic framework [*Cg*1⋯*Cg*2 = 3.643 (3) Å, where *Cg*1 and *Cg*2 are the centroids of the N3/N4/C3–C6 and C7–C12 rings, respectively; α = 3.8 (3)°, inter­planar distances = 3.466 (2) and 3.510 (2) Å, offset = 0.976 Å, symmetry code (i): −*x* + 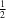, −*y* + 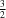, −*z* + 1].

## Database survey   

A survey of the Cambridge Structural Database (Version 5.38; Groom *et al.*, 2016 ▸) confirmed that the structure of the title complex has not been reported previously and revealed 41 Fe–Au CN-bridged frameworks supported axially by different co-ligands. There are 37 compounds with an octa­hedral FeN_6_ environment. The coordination spheres of such compounds are formed by pyridine-azine ligands, substituted pyridines, saturated and substituted pyrazines, and pyrimidine (Clements *et al.*, 2016 ▸; Arcís-Castillo *et al.*, 2013 ▸; Agustí *et al.*, 2008 ▸; Clements *et al.*, 2014 ▸, Kosone & Kitazawa, 2016 ▸; Niel *et al.*, 2003 ▸). Nine such compounds have a stable low- or high-spin state and another 28 are complexes with a switchable spin state. There are also four compounds with an environment formed by the N atoms of organic ligands and water O atoms. The only compound with an FeN_5_O environment contains a pyridine-based N-donor ligand (Xu *et al.*, 2014 ▸), while three compounds have an FeN_4_O_2_ octa­hedral geometry. The bidentate bridging organoselenium triazole ligand and two different pyridine-based ligands were used to obtain these latter complexes (Seredyuk *et al.*, 2007 ▸; Xu *et al.*, 2014 ▸).

## Synthesis and crystallization   

Crystals of the title compound were prepared by the slow diffusion method between three layers in a 10 ml tube. The first layer was a solution of K[Au(CN)_2_] (0.0058 g, 0.02 mmol) in water (2.5 ml), the second was a mixture of water/aceto­nitrile (1:2, 5 ml) and the third layer was a solution of 2-phenyl­pyrazine (0.0078 g, 0.05 mmol) and [Fe(OTs)_2_]·6H_2_O (0.0101 g, 0.02 mmol) (OTs = *p*-toluene­sulfonate) in aceto­nitrile (2.5 ml) with 0.3 ml of water. After two weeks, yellow crystals grew in the second layer; these were collected and maintained under the mother solution until measured.

## Refinement   

Crystal data, data collection and structure refinement details are summarized in Table 3 ▸. The hydrogen atoms were placed in their expected calculated positions (C—H = 0.93 Å) and refined as riding with *U*_iso_(H) = 1.2*U*_iso_(C). The idealized OH_2_ group was fixed using an AFIX 7 command that allowed the H atoms to ride on the O atom and rotate around the bond.

## Supplementary Material

Crystal structure: contains datablock(s) I, Global. DOI: 10.1107/S2056989019009678/su5503sup1.cif

Structure factors: contains datablock(s) I. DOI: 10.1107/S2056989019009678/su5503Isup2.hkl

CCDC reference: 1938914

Additional supporting information:  crystallographic information; 3D view; checkCIF report

## Figures and Tables

**Figure 3:**
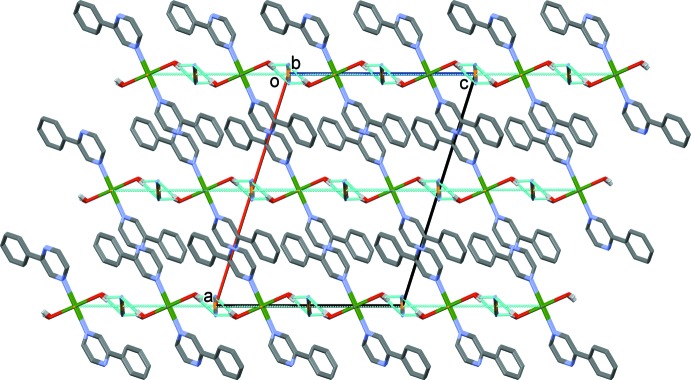
A view along the *b* axis of the crystal packing of the title compound. The O—H⋯N hydrogen bonds and Au⋯Au inter­actions are shown as dashed lines. For clarity, the C-bound H atoms have been omitted.

**Table 1:** Selected geometric parameters (Å, °)

Au1—C1	1.975 (7)	Fe1—N2	2.117 (6)
Au1—C2^i^	1.988 (7)	Fe1—O1	2.122 (4)
Au2—C13	1.988 (6)	Fe1—N3	2.223 (5)
Fe1—N1	2.107 (5)		
			
C1—Au1—C2^i^	180	N2—Fe1—N3	89.60 (10)
C13^ii^—Au2—C13	180	O1—Fe1—N3	90.09 (16)
N1—Fe1—N2	180	C1—N1—Fe1	180
O1—Fe1—O1^iii^	176.73 (19)	C2—N2—Fe1	180
N3—Fe1—N3^iii^	179.19 (19)	N1—C1—Au1	180
N1—Fe1—O1	91.63 (9)	N2—C2—Au1^iv^	180
N2—Fe1—O1	88.37 (9)	N5—C13—Au2	175.8 (7)
N1—Fe1—N3	90.40 (10)		

Symmetry codes: (i) 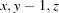; (ii) 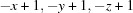; (iii) 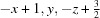; (iv) 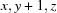.

**Table 2:** Hydrogen-bond geometry (Å, °)

*D*—H⋯*A*	*D*—H	H⋯*A*	*D*⋯*A*	*D*—H⋯*A*
O1—H1*A*⋯N5^v^	0.86	2.02	2.851 (6)	165
O1—H1*B*⋯N5^vi^	0.85	2.18	3.023 (6)	178

Symmetry codes: (v) 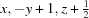; (vi) 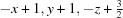.

**Table 3:** Experimental details

Crystal data	
Chemical formula	[AuFe(CN)_2_(C_10_H_8_N_2_)_2_(H_2_O)_2_][Au(CN)_2_]
*M*_r_	902.26
Crystal system, space group	Monoclinic, *C*2/*c*
Temperature (K)	293
*a*, *b*, *c* (Å)	18.5306 (13), 10.4541 (3), 14.2522 (9)
β (°)	107.509 (7)
*V* (Å^3^)	2633.0 (3)
*Z*	4
Radiation type	Mo *K*α
μ (mm^−1^)	11.70
Crystal size (mm)	0.3 × 0.3 × 0.1

Data collection	
Diffractometer	Rigaku Xcalibur Eos
Absorption correction	Multi-scan (*CrysAlis PRO*; Rigaku OD, 2015 ▸)
*T*_min_, *T*_max_	0.292, 1.000
No. of measured, independent and observed [*I* > 2σ(*I*)] reflections	7203, 3265, 2410
*R*_int_	0.031
(sin θ/λ)_max_ (Å^−1^)	0.667

Refinement	
*R*[*F* ^2^ > 2σ(*F* ^2^)], *wR*(*F* ^2^), *S*	0.038, 0.084, 1.04
No. of reflections	3265
No. of parameters	173
H-atom treatment	H-atom parameters constrained
Δρ_max_, Δρ_min_ (e Å^−3^)	1.37, −1.45

Computer programs: *CrysAlis PRO* (Rigaku OD, 2015 ▸), *SHELXS97* (Sheldrick, 2008 ▸), *SHELX2018* (Sheldrick, 2015 ▸), *OLEX2* (Dolomanov *et al.*, 2009 ▸), *Mercury* (Macrae *et al.*, 2008 ▸), *PLATON* (Spek, 2009 ▸) and *publCIF* (Westrip, 2010 ▸).

## supplementary crystallographic information

### Crystal data

**Table:**

[AuFe(CN)_2_(C_10_H_8_N_2_)_2_(H_2_O)_2_][Au(CN)_2_]	*F*(000) = 1680
*M**_r_* = 902.26	*D*_x_ = 2.276 Mg m^−^^3^
Monoclinic, *C*2/*c*	Mo *K*α radiation, λ = 0.71073 Å
*a* = 18.5306 (13) Å	Cell parameters from 2258 reflections
*b* = 10.4541 (3) Å	θ = 2.3–30.8°
*c* = 14.2522 (9) Å	µ = 11.70 mm^−^^1^
β = 107.509 (7)°	*T* = 293 K
*V* = 2633.0 (3) Å^3^	Plate, clear light yellow
*Z* = 4	0.3 × 0.3 × 0.1 mm

### Data collection

**Table:**

Rigaku Xcalibur Eos diffractometer	3265 independent reflections
Radiation source: fine-focus sealed X-ray tube, Enhance (Mo) X-ray Source	2410 reflections with *I* > 2σ(*I*)
Graphite monochromator	*R*_int_ = 0.031
Detector resolution: 8.0797 pixels mm^-1^	θ_max_ = 28.3°, θ_min_ = 2.3°
ω scans	*h* = −17→24
Absorption correction: multi-scan (CrysAlis PRO; Rigaku OD, 2015)	*k* = −7→13
*T*_min_ = 0.292, *T*_max_ = 1.000	*l* = −18→16
7203 measured reflections	

### Refinement

**Table:**

Refinement on *F*^2^	Primary atom site location: structure-invariant direct methods
Least-squares matrix: full	Secondary atom site location: difference Fourier map
*R*[*F*^2^ > 2σ(*F*^2^)] = 0.038	Hydrogen site location: mixed
*wR*(*F*^2^) = 0.084	H-atom parameters constrained
*S* = 1.04	*w* = 1/[σ^2^(*F*_o_^2^) + (0.0308*P*)^2^ + 0.2566*P*] where *P* = (*F*_o_^2^ + 2*F*_c_^2^)/3
3265 reflections	(Δ/σ)_max_ = 0.001
173 parameters	Δρ_max_ = 1.37 e Å^−^^3^
0 restraints	Δρ_min_ = −1.45 e Å^−^^3^

### Special details

**Table:**

Geometry. All esds (except the esd in the dihedral angle between two l.s. planes) are estimated using the full covariance matrix. The cell esds are taken into account individually in the estimation of esds in distances, angles and torsion angles; correlations between esds in cell parameters are only used when they are defined by crystal symmetry. An approximate (isotropic) treatment of cell esds is used for estimating esds involving l.s. planes.

### Fractional atomic coordinates and isotropic or equivalent isotropic displacement parameters (Å^2^)

**Table:**

	*x*	*y*	*z*	*U*_iso_*/*U*_eq_	
Au1	0.500000	0.51420 (2)	0.750000	0.03916 (11)	
Au2	0.500000	0.500000	0.500000	0.04886 (13)	
Fe1	0.500000	1.01389 (8)	0.750000	0.0304 (3)	
O1	0.4511 (3)	1.0197 (3)	0.8669 (2)	0.0440 (10)	
H1A	0.461953	0.949471	0.898996	0.066*	
H1B	0.474896	1.072647	0.910236	0.066*	
C12	0.2099 (3)	0.8548 (6)	0.3359 (4)	0.0498 (16)	
H12	0.177175	0.921680	0.337514	0.060*	
N3	0.3852 (3)	1.0154 (3)	0.6414 (3)	0.0372 (11)	
N1	0.500000	0.8124 (5)	0.750000	0.0381 (16)	
C1	0.500000	0.7031 (6)	0.750000	0.0347 (18)	
N4	0.2472 (3)	1.0277 (4)	0.4918 (3)	0.0487 (13)	
C3	0.3652 (3)	0.9310 (4)	0.5669 (4)	0.0366 (13)	
H3	0.398880	0.865572	0.565374	0.044*	
C9	0.3032 (4)	0.6538 (5)	0.3300 (4)	0.0556 (17)	
H9	0.335120	0.585689	0.328588	0.067*	
C13	0.4723 (4)	0.3157 (6)	0.4880 (4)	0.0519 (17)	
C7	0.2772 (3)	0.8418 (5)	0.4104 (4)	0.0355 (12)	
C6	0.3334 (3)	1.1041 (5)	0.6411 (4)	0.0453 (15)	
H6	0.343207	1.163949	0.691614	0.054*	
N5	0.4606 (3)	0.2077 (5)	0.4804 (3)	0.0572 (15)	
N2	0.500000	1.2164 (5)	0.750000	0.0465 (19)	
C10	0.2365 (4)	0.6684 (6)	0.2558 (4)	0.0558 (18)	
H10	0.222790	0.609994	0.204193	0.067*	
C8	0.3230 (3)	0.7399 (5)	0.4067 (4)	0.0448 (15)	
H8	0.368242	0.728887	0.456743	0.054*	
C5	0.2662 (4)	1.1083 (5)	0.5674 (4)	0.0518 (16)	
H5	0.231568	1.171427	0.570352	0.062*	
C11	0.1910 (4)	0.7682 (6)	0.2584 (4)	0.0553 (17)	
H11	0.146242	0.779119	0.207499	0.066*	
C4	0.2977 (3)	0.9364 (4)	0.4927 (4)	0.0336 (12)	
C2	0.500000	1.3240 (7)	0.750000	0.045 (2)	

### Atomic displacement parameters (Å^2^)

**Table:**

	*U*^11^	*U*^22^	*U*^33^	*U*^12^	*U*^13^	*U*^23^
Au1	0.0509 (2)	0.01133 (13)	0.0543 (2)	0.000	0.01452 (16)	0.000
Au2	0.0638 (3)	0.02992 (17)	0.0467 (2)	0.00972 (13)	0.00732 (17)	−0.00062 (12)
Fe1	0.0484 (7)	0.0125 (4)	0.0289 (5)	0.000	0.0094 (5)	0.000
O1	0.069 (3)	0.0265 (17)	0.035 (2)	0.0036 (17)	0.0132 (19)	−0.0003 (15)
C12	0.041 (4)	0.062 (4)	0.045 (4)	−0.003 (3)	0.011 (3)	−0.002 (3)
N3	0.045 (3)	0.025 (2)	0.041 (3)	0.0041 (18)	0.011 (2)	0.0012 (18)
N1	0.055 (5)	0.010 (2)	0.045 (4)	0.000	0.010 (3)	0.000
C1	0.038 (5)	0.026 (4)	0.041 (4)	0.000	0.013 (3)	0.000
N4	0.052 (3)	0.044 (2)	0.047 (3)	0.012 (2)	0.011 (2)	0.001 (2)
C3	0.043 (4)	0.023 (2)	0.042 (3)	−0.001 (2)	0.010 (3)	−0.003 (2)
C9	0.070 (5)	0.045 (3)	0.051 (4)	−0.004 (3)	0.018 (3)	−0.010 (3)
C13	0.071 (5)	0.041 (3)	0.040 (3)	0.012 (3)	0.011 (3)	−0.001 (3)
C7	0.036 (3)	0.034 (3)	0.037 (3)	−0.002 (2)	0.013 (2)	0.003 (2)
C6	0.058 (4)	0.034 (3)	0.044 (3)	0.011 (3)	0.015 (3)	−0.003 (2)
N5	0.083 (4)	0.040 (3)	0.047 (3)	0.006 (3)	0.017 (3)	−0.002 (2)
N2	0.075 (6)	0.018 (3)	0.041 (4)	0.000	0.010 (4)	0.000
C10	0.065 (5)	0.055 (4)	0.047 (4)	−0.023 (3)	0.016 (3)	−0.015 (3)
C8	0.045 (4)	0.043 (3)	0.041 (3)	−0.004 (2)	0.005 (3)	−0.008 (2)
C5	0.060 (4)	0.037 (3)	0.060 (4)	0.019 (3)	0.021 (3)	0.004 (3)
C11	0.042 (4)	0.079 (5)	0.037 (4)	−0.016 (3)	0.001 (3)	−0.006 (3)
C4	0.037 (3)	0.029 (2)	0.036 (3)	0.000 (2)	0.012 (2)	0.008 (2)
C2	0.067 (6)	0.019 (3)	0.046 (5)	0.000	0.014 (4)	0.000

### Geometric parameters (Å, º)

**Table:**

Au1—C1	1.975 (7)	N4—C5	1.329 (7)
Au1—C2^i^	1.988 (7)	N4—C4	1.333 (7)
Au2—C13^ii^	1.988 (6)	C3—C4	1.376 (7)
Au2—C13	1.988 (6)	C3—H3	0.9300
Fe1—N1	2.107 (5)	C9—C10	1.373 (7)
Fe1—N2	2.117 (6)	C9—C8	1.377 (7)
Fe1—O1	2.122 (4)	C9—H9	0.9300
Fe1—O1^iii^	2.122 (4)	C13—N5	1.149 (7)
Fe1—N3	2.223 (5)	C7—C8	1.374 (7)
Fe1—N3^iii^	2.223 (5)	C7—C4	1.492 (7)
O1—H1A	0.8564	C6—C5	1.368 (7)
O1—H1B	0.8479	C6—H6	0.9300
C12—C7	1.380 (6)	N2—C2	1.125 (8)
C12—C11	1.390 (7)	C10—C11	1.349 (8)
C12—H12	0.9300	C10—H10	0.9300
N3—C6	1.333 (6)	C8—H8	0.9300
N3—C3	1.344 (6)	C5—H5	0.9300
N1—C1	1.143 (8)	C11—H11	0.9300
			
C1—Au1—C2^i^	180	N3—C3—C4	123.4 (5)
C13^ii^—Au2—C13	180	N3—C3—H3	118.3
N1—Fe1—N2	180	C4—C3—H3	118.3
O1—Fe1—O1^iii^	176.73 (19)	C10—C9—C8	120.2 (6)
N3—Fe1—N3^iii^	179.19 (19)	C10—C9—H9	119.9
N1—Fe1—O1	91.63 (9)	C8—C9—H9	119.9
N2—Fe1—O1	88.37 (9)	N2—C2—Au1^iv^	180
N1—Fe1—O1^iii^	91.63 (9)	N5—C13—Au2	175.8 (7)
N2—Fe1—O1^iii^	88.37 (9)	C8—C7—C12	118.2 (5)
N1—Fe1—N3	90.40 (10)	C8—C7—C4	122.0 (5)
N2—Fe1—N3	89.60 (10)	C12—C7—C4	119.8 (5)
O1—Fe1—N3	90.09 (16)	N3—C6—C5	120.9 (5)
O1^iii^—Fe1—N3	89.89 (16)	N3—C6—H6	119.6
N1—Fe1—N3^iii^	90.40 (10)	C5—C6—H6	119.6
N2—Fe1—N3^iii^	89.60 (10)	C11—C10—C9	119.3 (6)
O1—Fe1—N3^iii^	89.89 (16)	C11—C10—H10	120.3
O1^iii^—Fe1—N3^iii^	90.09 (16)	C9—C10—H10	120.3
Fe1—O1—H1A	108.0	C7—C8—C9	121.2 (5)
Fe1—O1—H1B	109.9	C7—C8—H8	119.4
H1A—O1—H1B	100.6	C9—C8—H8	119.4
C7—C12—C11	120.1 (6)	N4—C5—C6	124.0 (5)
C7—C12—H12	120.0	N4—C5—H5	118.0
C11—C12—H12	120.0	C6—C5—H5	118.0
C6—N3—C3	115.3 (5)	C10—C11—C12	121.1 (6)
C6—N3—Fe1	123.0 (4)	C10—C11—H11	119.5
C3—N3—Fe1	121.6 (4)	C12—C11—H11	119.5
C1—N1—Fe1	180	N4—C4—C3	120.7 (5)
C2—N2—Fe1	180	N4—C4—C7	117.1 (5)
N1—C1—Au1	180	C3—C4—C7	122.3 (5)
C5—N4—C4	115.6 (5)		

Symmetry codes: (i) *x*, *y*−1, *z*; (ii) −*x*+1, −*y*+1, −*z*+1; (iii) −*x*+1, *y*, −*z*+3/2; (iv) *x*, *y*+1, *z*.

### Hydrogen-bond geometry (Å, º)

**Table:**

*D*—H···*A*	*D*—H	H···*A*	*D*···*A*	*D*—H···*A*
O1—H1*A*···N5^v^	0.86	2.02	2.851 (6)	165
O1—H1*B*···N5^vi^	0.85	2.18	3.023 (6)	178

Symmetry codes: (v) *x*, −*y*+1, *z*+1/2; (vi) −*x*+1, *y*+1, −*z*+3/2.
